# Exosomes as bio-inspired nanocarriers for RNA delivery: preparation and applications

**DOI:** 10.1186/s12967-022-03325-7

**Published:** 2022-03-14

**Authors:** Ala Amiri, Rafieh Bagherifar, Ehsan Ansari Dezfouli, Seyed Hossein Kiaie, Reza Jafari, Reihaneh Ramezani

**Affiliations:** 1 Faculty of Basic Sciences, Science and Research Branch, Islamic Azad University, Tehran, Iran; 2 Student Research Committee, Tabriz University of Medical Sciences, Tabriz, Iran; 3 Department of Nanobiotechnology, Faculty of Biological Sciences, Tarbiat Modares University, Tehran, Iran; 4 Nano Drug Delivery Research Center, Kermanshah University of Medical Sciences, Kermanshah, Iran; 5 Solid Tumor Research Center, Cellular and Molecular Medicine Institute, Urmia University of Medical Sciences, Shafa St, Ershad Blvd., P.O. Box: 1138, 57147 Urmia, Iran; 6 Department of Biomedical Sciences, Women Research Center, Alzahra University, 1993893973 Tehran, Iran; 7 Department of Health Technology, Technical University of Denmark, Lyngby, Denmark

**Keywords:** Drug delivery system, Nanocarrier, Extracellular vesicle, Exosome, RNA transport

## Abstract

Nanocarriers as drug/biomolecule delivery systems have been significantly developed during recent decades. Given the stability, reasonable delivery efficiency, and safety of nanocarriers, there are several barriers in the fulfillment of successful clinical application of these delivery systems. These challenges encouraged drug delivery researchers to establish innovative nanocarriers with longer circulation time, high stability, and high compatibility. Exosomes are extracellular nanometer-sized vesicles released through various cells. These vesicles serve as nanocarriers, possessing great potential to overcome some obstacles encountered in gene and drug delivery due to their natural affinity to recipient cells and the inherent capability to shuttle the genes, lipids, proteins, and RNAs between cells. So far, there has been a lot of valuable research on drug delivery by exosomes, but research on RNA delivery, especially mRNA, is very limited. Since mRNA-based vaccines and therapies have recently gained particular prominence in various diseases, it is essential to find a suitable delivery system due to the large size and destructive nature of these nucleic acids. That's why we're going to take a look at the unique features of exosomes and their isolation and loading methods, to embrace this idea that exosome-mediated mRNA-based therapies would be introduced as a very efficient strategy in disease treatment within the near future.

**Graphical Abstract:**

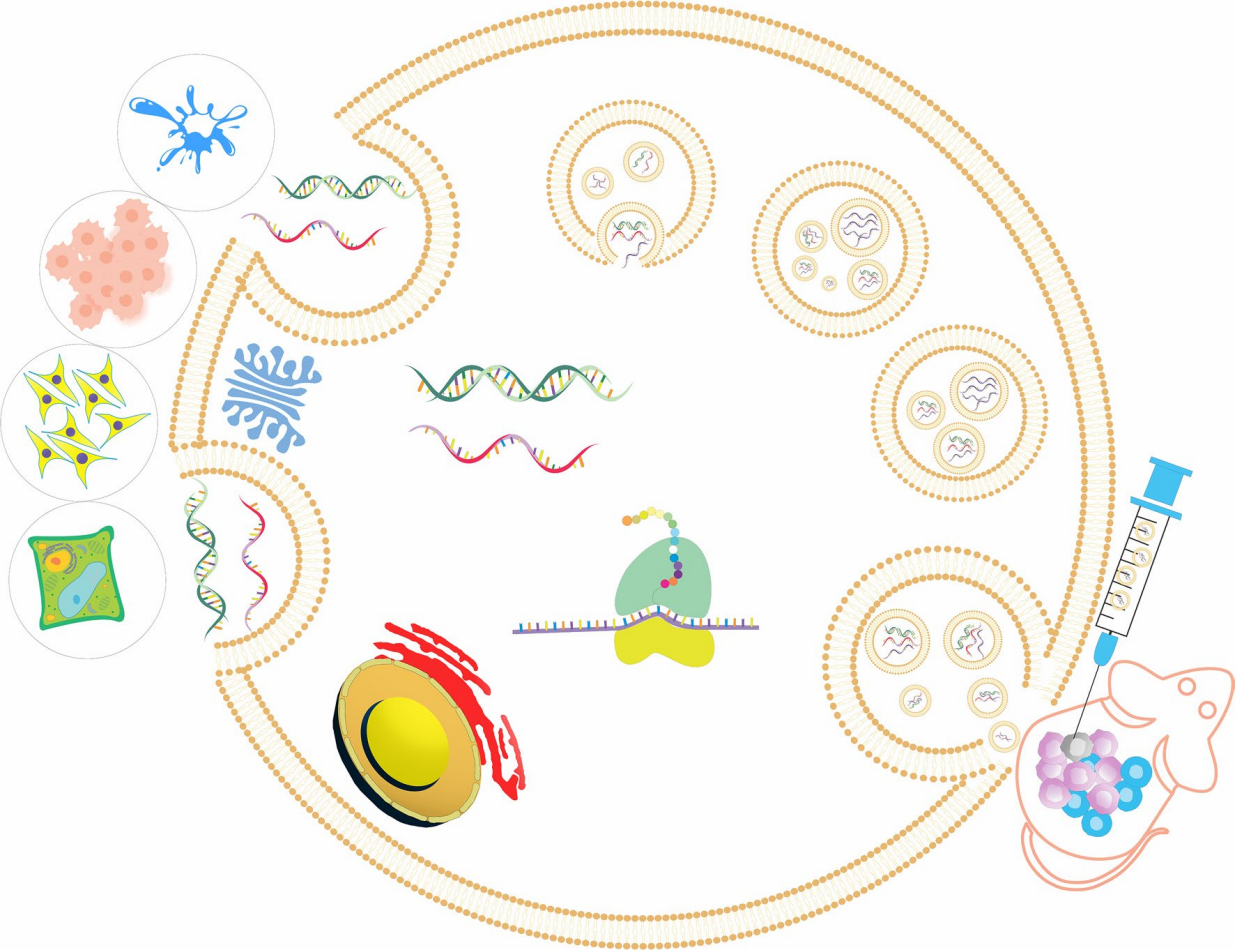

## Introduction

Exosomes are extracellular membranous vesicles (EMVs) possessing the special compositions of DNA, coding RNAs, non-coding RNAs, lipids, and proteins [1–4]. These endocytic membranes-derived vesicles can deliver various signals to target cells, thereby mediating new cell-to-cell communication mechanisms [3]. In -molecular-scale studies, exosomes possess phospholipases and lipid-related proteins, proteins participating in the biosynthesis of multi-vesicular bodies (MVBs) (TSG101, Alix), proteins involved in transport and fusion of membranes (flotillin, GTPases, annexins), heat shock proteins (i.e., Hsp90, Hsc70), and tetraspanins (CD82, CD81, CD63, CD9) [5]. Until now, a total of 4500 various proteins have been detected with exosomes, often through mass spectrometry (MS) [6], likely serving as cargos for intercellular communications. In addition to the above-mentioned proteins, the membranous vesicles are enriched in special raft-related lipids, like phosphoglycerides with saturated fatty-acyl and long chains, sphingolipids, ceramide, and cholesterol (mostly B lymphocytes-derived exosomes) [7, 8].

The previous reports have proven that the exosomes derived from various fluids in the body [9, 10] and various cell lines [11] possess RNA molecules, especially mRNAs and miRNAs, which can be translated into proteins or regulate protein expression in the recipient cells, respectively [12–15]. Current deep-sequencing studies have revealed that the membranous vesicles possess a variety of RNA cargoes comprising full-length RNAs with 25–250 nucleotides in lengths like tRNA and miRNA, as well as fragments of long RNA like rRNA, mRNA, even though mRNA molecules are presented in full-length types [16–21].

Exosomes play multi-pronged roles in the tissues or cells of origin to facilitate movement of pathogens such as viruses [22] and prions from one cell to another cell [23], inducing tumorigenesis [24], coagulation [25], inflammation [26], angiogenesis [4], programmed cell death [27], antigen presentation [3, 28], improvement of the immune responses, and deletion of debris molecules [29]. Interestingly, exosomes have been proved to enable signaling and cell-to-cell communications and deliver macromolecular messages such as proteins and RNAs [1]. Interest towards these membranous vesicles ranging from in vivo roles to further uses like their application in therapeutics, biomarker development, and diagnostics (according to the assay of their protein and RNA extent) has substantially increased in the past decades [22, 26, 29].

Owing to their potential application in translational research, these vesicles have fascinated a lot of fieldwork attention on their functions in various diseases and health [8, 26, 30–32]. These EMVs, especially mesenchymal stem cells (MSCs)-derived exosomes play an important role in kidney diseases, wound healing, liver diseases, neurodegenerative and autoimmune disorders, diabetes, spinal cord injury, and other diseases. Moreover, exosomes have attracted a great deal of attention as novel bio-carriers for gene and drug delivery because of the advantages such as less toxicity, the ability to cross biological barriers, and evade mononuclear phagocytic system (MPS) compared to synthetic nanoparticles [33]. Thus, highly efficient procedures for loading exosomes with drugs or biomolecules are prerequisites to achieving breakthroughs in the future [17, 22]. Although several studies have elucidated the detection, isolation, and characterization of exosomes along with their uses in drug delivery [1, 2, 4, 25, 26], the preparation and application of exosomes as the bio-inspired nanocarriers in RNA-based therapeutics have not been explored. In this study, the cutting-edge advances in the latest studies for the isolation and production of exosomes along with their clinical application in the delivery of RNA molecules, have been discussed. We revealed that exosomes with their unique properties and safety could open a new avenue for researchers in the development of efficient mRNA delivery system. Therefore, the establishment of large-scale RNA-loaded exosomes for clinical applications should be given further attention.

## Biogenesis

Exosome biogenesis starts with the generation of early endosome through inward budding of the cellular membrane followed by second budding of the endosomal membrane. The second budding leads to the production of late endosome. These endosomes including intraluminal vesicles (ILV), are known as multivesicular bodies. These bodies either follow the endocytic pathway for the exosome generation or fuse with lysosome to degradation [25]. During inward budding of the endosomal membrane, proteins, miRNA, mRNA, and DNA fragments are incorporated into the forming vesicles by very specific protein complex. Finally, by fusion of the ILV with plasma membrane the exosomes are released into extracellular space [26].

## Sources of exosomes

The term "exosome" was first presented in the 1980s for vesicles released by a cell line having ectoenzyme activities [34]. In fact, it refers to the vesicles secreted during the differentiation of reticulocytes [35], whereas, the EMVs are reported to be secreted through dendritic cells (DCs) and B lymphocytes via a similar path [24]. In the latter years, several cell kinds of nonhematopoietic/hematopoietic origin, like oligodendrocytes, intestinal epithelial cells, Schwann cells, neurons, platelets, cytotoxic T cells, and mast cells have been documented to release the EMVs [29, 36]. Moreover, the vesicles have been found in the various fluids of the body, such as bile [37], cerebrospinal fluid [28], amniotic fluid [38], ascites fluid [39], breast milk [40], urine [41], semen [42], saliva [43], and blood [44].

### Stem cell-derived exosomes

Recently, efforts to repair human tissues by stem cells (SCs) have attracted considerable attention in regenerative medicine [23]. Many SCs like induced pluripotent SCs (iPSCs), embryonic SCs (ESCs), and MSCs with considerable potential in cell proliferation and differentiation have been used to repair human tissues [4, 13, 45]. SCs were found to discharge many products in a paracrine manner that results in their related influences. These products consist of extracellular vesicles, cytokines, and various growth factors [23]. EMVs released through SCs are commonly pointed out as exosomes, shedding vesicles, microparticles, cell-derived vesicles, and microvesicles [46]. The exosomes secreted from SCs have exhibited considerable potential in cell-free regenerative medicine. For example, Gibbings et al. [47] suggested that the exosomes derived from SCs can package particular miRNAs for regulating the various cell processes. This provides an opportunity for EMVs-based remedial approaches in musculoskeletal diseases because the miRNA molecules have essential functions in the prevention and progression of disorders.

### Dietary sources-derived exosomes

EMVs and their cargos can be isolated from dietary resources, like milk. These vesicles and cellular glycoproteins are imperative for intestinal uptake. A considerable fraction of milk-derived vesicles accumulates in the brain tissue [47]. The part of milk-derived EMVs that gets away from absorption elicits alteration in microbial populations in the gastrointestinal tract [47]. Dietary discharge of EMVs and their cargos leads to a loss of circulating RNAs and induces some properties, like the changed immune responses, loss of fecundity, increased purine metabolites, and loss of cognitive performance [23]. Overall, the exosomes derived from milk meet the definition of bioactive ingredients in foods. In an applied study, Reif et al. [48] presented evidence that milk-derived exosomes possess a various biological efficacy on normal fetal colon epithelial cell in contrast to colon tumor cell in a miRNA-dependent manner. Thus, they suggested that the positive efficacy of exosome on normal cell without affecting tumor cell can present a promising aspect of its safety when regarding its application as a nutritional supplement to infant formula.

### Plant cell-derived exosomes

Exosomes from non-animal organisms remain underestimated, and our understanding of them is still expansible, albeit animal-derived exosomes are moving increasingly into interest areas of research [23]. This is due to the lack of a practicable and easy visualization, purification, and isolation procedure. The exosomes from *Viscum album* L., *Vinca minor* L., and *Nicotiana tabacum* L., were isolated by differential centrifugation supplemented with agarose gel electrophoresis. It is claimed that the combination of electrophoresis with differential centrifugation can improve the isolation of exosomes [49]. Woith and Melzig purified exosomes from the dried material of plants, successfully. Further, exosomes can be separated from dye excess and small charged impurities because the contaminants can pass the gel matrix, whereas exosomes remain within the wells and do not enter the gel. [23, 49, 50].

### Cancer cell-derived exosomes

According to the fact that cancerous cells release many more EVs in contrast to normal cells, it was found that (1) the active secretion of exosomes possesses functional senses, although it is unknown whether they are cancer-suppressing or promoting [51]; (2) the exosomes can be utilized as markers for cancer diagnosis [23]. Interestingly, it was detected that the interactions caused by the exchange of exosomes among tumor stroma and cancer cells can promote the transfer of oncomicroRNAs (e.g., miR375, miR16, miR15, miR1, and let7) as well as oncogenes (e.g., LMP-1, Melan-A/Mart-1, HER2, CEA, and β-catenin) from one cell to another, resulting in new programming in target cells [4]. The functional roles and molecular compositions of cancerous cell-derived EMVs in response to therapy, metastasis, and tumorigenesis are gradually deciphered [23]. The potential therapeutic approaches and latest achievements in the terms of cancer cell-derived exosomes were elucidated by Kalluri and LeBleu [4].

## Isolation and purification methods for exosomes

**Fig. 1:**
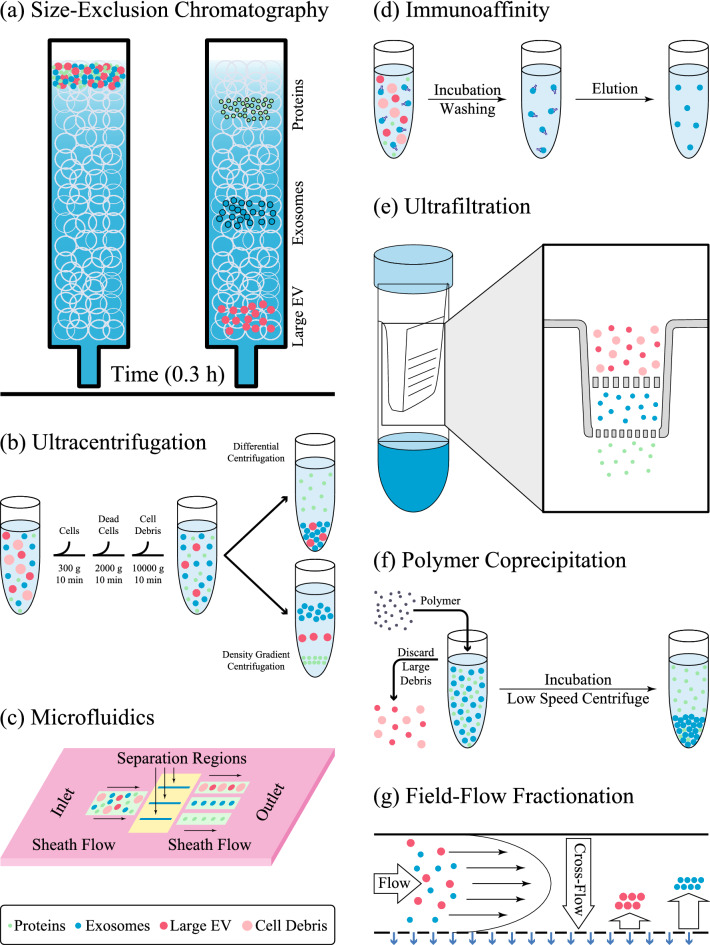
Schematic diagram of isolation methodology of exosomes. **a** Size exclusion chromatography which separates particles based on size, is one of the most common methods for obtaining a large volume of exosomes due to the lack of protein contamination and the ability to purify the exosome on a large scale. **b** Ultracentrifuge separation, despite being dependent on expensive equipment, has been widely used to isolate exosomes based on size and sedimentation properties or density in sucrose gradients. **c** Microfluidics-based methods rely on physical properties such as size and density, or chemical properties such as binding to exosome surface antigens. **d** In immunoaffinity methods, exosomes are captured based on their specific binding to antibodies or magnetic nanoparticles. As a result, the surface chemical properties are critical in these techniques. **e** In ultrafiltration, the particles are centrifuged through the filter and separated based on the pore size of the filter. **f** In the polymer co-percipitation method, based on steric exclusion, particles are gathered by PEG to form clumps that can be easily precipitated by low-speed centrifugation. **g** In field flow fractionation, particles accumulate at different position of the membrane depending on their size. Separation occurs when the diffusing and cross-flow forces are balanced

With the rapid progress in technology and science, many methods have been developed for exosome isolation in good purity and quantity. Each method exerts a specific characteristic of exosomes, like their surface proteins, size, shape, and density, to facilitate their isolation [22]. Some of the isolation procedures along with their mechanism of isolation and drawbacks and profits of the method, are summarized in Table 1 and Fig. 1. There are no efficient methods for exosome isolation, and the existing methods either encounter the problem of contamination with proteins and non-exosomal vesicles or are very expensive due to requiring special equipment [52]. The affinity-based methods are very pure; however, their high costs and low yields have prevented its extensive use in exosome purification. Researchers believe that a combination of two methods can boost the efficiency and purity of the isolated exosomes [53]. The field flow fractionation methods have received special attention due to their scalability and isolation of specific subpopulations of exosomes [54]. However, they require further improvement for use in clinical applications.

**Table 1:** Isolation methods for exosomes

Isolation procedures	Mechanisms	Drawbacks	Profits	Refs.
Size-exclusion chromatography	Size	Lack of specificity	High recovery	[58]
Ultracentrifugation	Size and sedimentation properties or density	Low recovery, large sample volume, long duration	Gold standard, high purities	[30]
Microfluidics	Size and density	Complexity of device, low throughput	Easy to automate, convenient, low cost, and fast	[59]
Immunoaffinity	Affinity purifications	Restricted use, low yield, high cost	High purity and specificity	[60]
Ultrafiltration	Molecular weight and size	Difficult scaling	High recovery, fast/simple operation	[61]
Polymer coprecipitation	Surface charges	Complex scaling, lack of specificity	User-friendly and easy processing	[62]
Field-flow fractionation	Molecular weight and size	Needs fractionation equipment, long duration	Wide variety of eluents, broad separation range	[54]

Procedures for the purification and preparation of extracellular vesicles in large amounts include exosome precipitation [55], ultrafiltration [56], sucrose density gradient ultracentrifugation, and differential ultracentrifugation [30]. In a study in which large-scale mRNA-loaded exosomes were prepared, two differential ultracentrifugation and density gradient ultracentrifugation methods were used to purify the exosomes. It was observed that the rate of mRNA recovery is similar in both procedures. At the same time, the chemicals used in the density gradient filtration method may not be removed entirely. Furthermore, more RNAs were concentrated in the exosome fractions when differential ultracentrifugation was used. Hence, differential ultracentrifugation was chosen as a preferable method to purify the exosomes [57].

## Methods for loading RNA into exosomes

Exosomes are natural intercellular transporters of RNAs that are responsible for different roles in vivo. Because of their intrinsic features, these EMVs can present a more effective procedure for transfecting RNAs in gene therapy than classic delivery vehicles of nucleic acids [63]. Despite many benefits, the exosome-based RNA delivery is restricted since generating adequate amounts of RNA-loaded exosomes for in vivo application is technically challenging [45, 64]. First, only a few cell sources have been observed to release an adequate quantity of exosomes that are needed for clinical translation [27, 65, 66]. Second, to produce a clinical dose of the EMVs, a great deal of cell culture should be incubated for multiple days, followed by RNA isolating and loading before the ultimate gene-enriched vesicles could be achieved. Although post-insertion of siRNA and shRNA plasmid into exosomes through electroporation technique has shown more remedial efficacies than synthetic nanocarriers in repressing oncogenic targets in the pancreatic cancer preclinical models [66], loading of large RNAs like mRNAs into exosomes remains challenging technically [67]. Herein, our group discussed several approaches to incorporate RNA molecules into exosomes for therapeutic applications and transcriptional manipulations. These approaches include guidance of signature sequences, electroporation, transfecting donor cell, transfection with specific reagents, producing hybrid exosome-liposome and cellular nanoporation [4, 63, 68]. In some loading methods such as electroporation, liposome-exosome hybrids, and transfection with specific reagents, exosomes are first purified from the appropriate sources, and RNA molecules are then loaded into its structure. However, in other techniques such as the guidance of signature sequences and transfecting donor cells, the cells are employed to load nucleic acids by engineering the exosome-producing cells. Therefore, the exosomes secreted from these cells will contain the desired RNA molecules.

### Guidance of signature sequences

The actively packaging of nucleic acids as one of the methods for loading nucleic acids into the EMVs has attracted the researcher's attention [63]. Active packaging by proteins binding to special RNAs was used to generate exosomes in engineered mammalian cells [22]. By transfecting and engineering exosome-producing cells, researchers have been able to use the cellular potential to load catalase mRNA into exosomes. According to their results, exosomes can deliver the mRNA cargo to brain cells and significantly attenuate the effects of neurotoxicity and neuro-inflammation in Parkinson’s disease [69]. The conserved sequence of exosome-enriched RNAs (eRNAs) was also presented as a procedure to perform active packaging. The authors proposed that exosome-enriched RNAs might possess a special common sequence that aims RNAs into exosomes as *cis*-elements. The results revealed that only three motifs are potential signature sequences. The findings help us understand the selective targeting of candidate mRNA to the exosome for future therapeutic research [63, 70].

### Electroporation

For a long time, electroporation has been known as an efficient and fast procedure to load siRNAs and miRNAs into the extracellular vesicles. Jing-Hung Wang et al. demonstrated that the electroporation method is applicable for loading siRNA into exosomes. Simultaneously, it is not adequate for mRNA loading because less mRNA entered the electroporated exosomes compared to the non-electroporated ones, indicating that the mRNA remained not internalized. Therefore, they used a new method involving transfecting 293FT cells with a plasmid type to insert HChrR6 mRNA into exosomes [71]. The researchers fused the neuron-specific peptide RVG with exosome membrane protein Lamp2b [60] in another study. The siRNAs were loaded into purified exosomes to make these extracellular vesicles target neuron cells through the electroporation technique [22].

Moreover, Alvarez-Erviti et al. reported that exosomes loaded with BACE1-targeted siRNA prepared via electroporation method can be delivered to the patient's brain to treat Alzheimer's conditions [65]. Overall, exosome transfection with nucleic acids through electroporation is straightforward, but it is not high potent and efficient. This procedure needs purification and separation of extracellular vesicles before and after the transfection process. High-speed centrifugation might destroy exosomes and decrease the quality of samples. It was also found that repeated purification can lead to loss of exosomes [2, 63]. In fact, electroporation is limited to some RNAs, while miRNAs, shRNAs, and mRNAs cannot be loaded to exosomes with this method [72].

### Transfecting donor cell

In this method, the parental cells are transfected with siRNAs and miRNAs, which subsequently release into the exosomes via the parental cells [63]. It was found that the secretion of exosomes from fat SCs overexpressing miR126 takes part in a remedial task in relieving myocardial ischemic acute injuries [73]. The exosomes released by HEK293T cells transfected with the miR-214 inhibitor showed the ability to reverse the cisplatin resistance of gastric cancers [71]. In another study, SGC-7901 and HEK293T cells transfected with siRNA and the released HGF siRNAs loaded exosomes were found to be able to suppress the migration and growth of cancer/vascular cells [62]. Moreover, Sun et al., transfected HEK293T cells with the IRESLuc reporter plasmid and demonstrated that about 50–70 mRNA copies loaded per 100 exosomes [74]. In Vakhshiteh's study, it was found that miR-34a-loaded exosomes after overexpression of mesenchymal stem cells were more efficient in inhibiting the proliferation of breast carcinoma cells than liposomes [75]. This method, however, is restricted by inadequate packaging, lacking specificity, and cytotoxicity [63].

### Transfection with specific reagents

Transfection with specific reagents was proposed as an alternative approach for loading nucleic acids into exosomes. In an effort, exosomes were obtained from the HeLa cell line through the ultracentrifugation technique. The lipofectamine-combined siRNA AF488 was then incubated with the extracellular vesicles for 25 min. The mixture was washed and ultrafiltered after the transfection process. The siRNA-loaded extracellular vesicles were cultured together with the target cells for one day. Eventually, the authors suggested that the exogenous siRNA molecules can be introduced to the recipient cell by using transfection with specific reagents. The main disadvantage of this method is that the exosome cannot be separated from lipofectamine and it is not clear whether the transfection is the result of lipofectamine or the exosome [76, 77].

Recently, System Biosciences Co. developed a commercial Kit for exosome delivery, namely Exo-Fect™ Exosome Transfection Kit [75]. This company claimed that the researchers can turn exosomes into delivery vehicles with Exo-Fect, enabling the insertion of small molecules, DNAs (including plasmids), and RNAs into isolated exosomes. Their kit has several features, including (1) introduction of various biomolecules directly into isolated exosomes: metabolites and other small molecules; DNAs including plasmids; RNAs including mRNAs, miRNAs, and siRNAs; (2) easy-to-use with a straightforward and fast loading procedure [75].

### Producing hybrid exosome with liposome

Directing the CRISPR/Cas to the host cells is vital for genome editing in vivo. The performance of packaging nucleic acids within exosomes, however, is low. Because liposomal particles are made artificially, and drugs are feasibly loaded into these structures, they are widely used in the pharmaceutical industry nowadays. Although the liposomal particles are applied as nano-carriers, the main challenge with these nanovesicles is their low efficiency in cell entry and drug delivery, which exosomes are experts to do. However, the amount of loaded drugs and nucleic acids is meager in the exosome. In this regard, the researchers tried to form a new structure, called the liposome-exosome hybrid, by simply incubating two nanoparticles and inducing membrane fusion to overcome the difficulty of using either of these nano-carriers alone [78–80]. Lin et al. developed hybrid exosomes-liposomes and they found that these mixed nanoparticles (NPs) can package the CRISPR-Cas9 expression vector as a large plasmid efficiently. The authors demonstrated that the MSCs can endocytose and express these hybrid NPs. The liposomes had no ability to transfect the SCs alone. Thus, the hybrid NPs of exosome-liposome were proved to deliver CRISPR-Cas9 systems in the SCs efficiently, proposing a potent application of this method in mRNA encapsulation in the future [81].

### Cellular nanoporation: accelerating exosome release

The cellular-nanoporation procedure was firstly established for the large-scale production of exosomes containing therapeutic mRNA molecules [57]. Yang et al. transfected different cells with plasmids and provoked the cells with focal/transient electrical motivations, which induce the secretion of EMVs harboring transcribed mRNA. This procedure can generate up to 55-fold more vesicles and achieve up to 1000-fold increment in exosomal mRNA transcripts, even from cells with a low level of EMVs release and without genetic modification of the source cells [57]. Therefore, the cellular nanoporation approach enables the application of the vesicles as a potential mRNA carrier for uses that need transcriptional manipulations.

## Therapeutic applications of exosomes

### Exosomes with intrinsic therapeutic activity

The substantial remedial and biomedical potential of exosomes was not forecasted when they were at first detected in the 1980s [3, 22]. These membranous vesicles take part in intercellular communications through delivering their content, such as proteins, mRNAs, and miRNAs to target cells, with or without direct contact among cells. In addition, exosomes affect pathological and physiological processes. Owing to the important properties, exosomes can enhance motor and neural functions in the nervous system, allow multiple intravenous dosing without any side effects, cross the blood–brain barriers, and decrease inflammation. These membranous vesicles play a crucial role in diagnosis and prognosis of many pathological conditions like numerous cardiopulmonary disorders, kidney and liver disease, neurodegenerative disorders, and cancer [82].

Current researches have indicated that exosomes derived from different sources are a new remedial tool.

#### MSCs-derived exosomes

The MSCs-derived exosomes have been assayed in disease models like diabetes mellitus, renal disease, dermatological, gastrointestinal, hepatic, musculoskeletal, neurological, cardiovascular, and respiratory disorders [32]. These vesicles promoted tissue regeneration by improving extracellular matrix remodeling. They prevented the production of the pro-inflammatory cytokines resulting in anti-inflammatory effects [83]. MSCs-derived exosomes were found to have the cytoprotective and immunomodulatory activity of their parent cells [84]. It was shown that exosomes obtained from MSCs could enhance cognition by protecting oxidative damages in astrocytes and neurons in diabetic animals suffered from cognitively impaired conditions [85]. Administration of MSCs-derived exosomes can also decrease infarct sizes in myocardial reperfusion/ischemia models [86, 87]. MSCs-derived exosomes with GATA-4 overexpressing reduced cardiomyocyte apoptosis and increased cardiomyocyte survival [88]. Bone marrow MSCs-isolated vesicles were found to protect against different kinds of diseases like brain injury, hypoxia-derived pulmonary hypertension, and ischemia injury [31, 86, 89]. These MSCs-derived exosomes showed neurorestorative influences, such as an increment in myelin and axon density in rats with diabetes II and a decrement in blood–brain barrier haemorrhage and leakage [90].

Similar to MSCs-derived vesicles, exosomes secreted from cardiac progenitor cells, ESCs, and iPSCs possess therapeutic effects [91]. The intravenous injection of urinary SCs-isolated exosomes can decrease apoptosis and urinary albumin, and increase the glomerular endothelial cell growth in rats treated with streptozotocin, demonstrating exosomes can be a new procedure to treat diabetic nephropathy [92].

#### Milk-derived exosomes

Milk-derived exosomes have some favorable characteristics, including staying completely intact during gastric digestion, accessible entrance into the blood circulation through endocytosis in the gastrointestinal tract, transferring their protein and miRNA contents to the immune cells, and subsequently regulating immune response and growth [93]. Moreover, bovine milk-derived exosomes can attenuate arthritis [94]. Milk exosomes can also be employed as carriers for oral drug delivery. Agrawal et al. reported that oral administration of Paclitaxel-loaded exosomes showed less toxicity and side effects than intravenous administration [95].

#### DC-derived exosomes

Intradermal/subcutaneous injections of DC-derived exosomes resulted in stabilizing of objective tumor response or another disease [96]. DC-derived vesicles containing tumor antigens can induce cancer-specific T cell responses [97]. Exosomes derived from dendric cells (DCs) showed a more remarkable ability to produce anti-tumor antibodies than DC cells. Due to the presence of MHC class I / II in the membrane structure of DC-derived exosomes, researchers believe that these vesicles would be used as a very efficient platform in vaccination in the near future [98].

#### Tumor-derived exosomes

Tumor cell-derived exosomes were documented to induce immunosuppression by stimulating regulatory T cells and immunosuppressive myeloid-derived suppressor cells, suppressing DC differentiation and NK cell cytotoxicity, and inducing T cell apoptosis [99, 100]. While, exosomes that derived from tumor cells under stress condition could induce antitumor immune responses because they contain more HSPs (heat-shock proteins) in addition to tumor antigens. For instance, B16BL6 murine melanoma-originated exosomes incubated with melanoma antigens could induce particular T-cell responses and suppress cancer growth [101].

### Exosomes as drug carriers

Exosomes have been potentially used as drug carriers in various diseases and cancers since their discovery due to their unique properties such as stability, tumor targeting, and biocompatibility [102]. Withaferin, doxorubicin, and paclitaxel-loaded exosomes released these agents slowly and suppressed the lung tumor growth in vivo and A549 lung tumor in vitro*,* effectively [103]. Furthermore, when these agents were loaded into exosomes, they indicated a lower IC50 value than when they were used as free drugs. EL-4 mouse lymphoma-isolated exosomes loaded with curcumin suppressed pro-inflammatory cytokines, like IL-6 and TNF-α [104]. Lower levels of cardiotoxicity along with the higher concentration of intracellular doxorubicin were obtained in doxorubicin-loaded exosomes in contrast to its systemic administration [105]. Similarly, cytotoxicity and high neoplastic tropism were recorded in MDR pulmonary metastases in pursuit of using exosomes encapsulated with Taxol [61].

### Exosome-mediated RNA therapeutics

RNAs are large biomolecules and problematic to deliver in vivo. Some delivery systems like cationic polymer-based particles [106], dendrimers [107], and cationic liposomes [108, 109] have been proposed. These carriers, however, are not appropriate for application in clinical practices, because of off-target issues and inadequate stability and safety [110]. Exosomes as lipid-bilayer enclosed, nanometer-sized extracellular vesicles serve as potential nucleic acid carriers owing to their natural affinity to recipient cells and inherent capability of shuttling DNA and RNA between cells [4]. Until now, exosome-based nanocarriers delivering a variety of RNAs—including mRNA, siRNA, and miRNA—have been established to treat different diseases and various cancers [67] (Tables 2 and 3).

**Table 2:** Exosome-based RNAs delivery for treating neurological diseases

Neurological diseases	Exosome-based RNA delivery systems	Therapeutic results	Refs.
Huntington’s disease	HEK 293-cells derived exosomes loaded with MiR124	Decrease in expression of the target gene; no improvement was recorded following exosomes treatment loaded with miR-124	[89]
Brain infarct	murine BM-MSCs-derived exosomes loaded with MiR124	Protecting against ischemic injury through robust cortical neurogenesis; miRNA specific effect on the ischemic regions	[68]
Morphine relapse	HEK 293 T cells-derived exosomes loaded with MOR siRNA	Specific delivery of siRNA to the brain; down-regulation of the level of MOR protein and mRNA	[127]
Alzheimer’s disease	mouse DC-derived exosomes loaded with siRNA	Knockdown of target mRNA and protein	[65]

**Table 3:** Exosomes-based RNAs delivery for treating cancers

Tumor type	Exosome-based RNA delivery systems	Therapeutic results	Refs.
Glioblastoma multiforme	MSC-derived exosomes loaded with anti miR9	Reversing of chemoresistance of glioblastoma multiforme cells	[128]
Glioma	MSC-derived exosomes loaded with MiR146	Inhibition of glioma xenograft growth in glioma mice	[118]
Hepatocellular carcinoma	HLSCs-derived exosomes loaded with miRNA	Inhibition of the growth of hepatocellular carcinoma in mouse	[129]
Breast cancer	HEK293 cells-derived exosomes loaded with let-7a miRNA	Specific delivery of Let-7a miRNA to breast cancer tissue and strong inhibition of tumor growth	[72]
Breast tumor	Breast cells-derived exosomes loaded with HchrR6 mRNA	Inhibition of growth of HER2-positive human breast cancer	[71]
Glioma mouse model	Glioma cells-derived exosomes loaded with bEND mRNA	mRNA-loaded exosomes restored tumor-suppressor activity	[57]
U-87 Glioblastoma	bEND.3 cells-derived exosomes loaded with VEGF siRNA	Effective suppression of the aggregation of xeno-transplanted cancer cells; knockdown of VEGF in glioblastoma-astrocytoma cells	[68]
Mouse sarcomas	Mouse fibroblast L929 cells-derived exosomes loaded with TGF-β1 siRNA	Suppression of the growth and metastases of tumor in mice; strong inhibition of TGF-β1 expression	[130]
Breast cells	Endothelial exosomes loaded with siRNA against luciferase	Prevention of luciferase expression in the target cell	[131]
HeLa cells	HT1080 human fibrosarcoma/Hela cells-derived exosomes loaded with siRNA	Remarkable knockdown of the target protein	[76]
Pancreatic cancer	BM-MSC exosomes loaded with galectin-9 siRNA	effective innate and adaptive anti-PDAC immunotherapy upon disruption of galectin-9/dectin 1 axis	[132]
Colon cancer	HT-29 and SW480 derived exosomes loaded with miR-375-3p mimic	reverse epithelial mesenchymal transition (EMT) process of colon cancer cells	[133]

#### Exosomes as carriers for siRNA delivery

siRNA is a biological macromolecule with a polyanionic nature that leads to poor passive uptake. Furthermore, due to the possibility of degradation by nucleases, it is not possible to inject naked siRNA into the systemic circulation. Exosomes have been shown to be an ideal nanocarrier for siRNA encapsulation due to their structure consisting of a hollow, aqueous core surrounded by a phospholipid bilayer, as well as their stability in the blood and inherent targeting properties [111]. El-Andaloussi et al. suggested an approach to utilize exosomes to deliver siRNA molecules in vivo/in vitro to the mouse brain that resulted in a promising output [112]. Moreover, Alvarez-Erviti et al. utilized murine self-derived dendritic exosomes for delivering BACE1 and GAPDH siRNAs across the mouse blood–brain barrier. They observed that the BACE1 and GAPDH gene-loaded exosomes greatly suppressed the gene expression and β-amyloid production in the mouse brain following intravenous injection [65]. Wahlgren et al. observed that human plasma-isolated exosomes loaded with siRNA successfully targeted lymphocytes and monocytes and eventually silenced the MAPK1 gene. They also demonstrated that exosomes loaded with siRNA co-localized in the target cell cytoplasm. Further, RAS52 and RAD51 siRNAs-enriched exosomes induced the death of fibrosarcoma cells, showing that exosomes can be utilized as a vector in the gene therapy based on RNAi. It should be noted that, in the in vitro studies, the target cells take up the EMVs easier than in vivo [76, 113]. Recently researchers illustrated that silencing the CTGF gene via siRNA-loaded MSCs-derived exosomes effectively reduced the inflammation and neuronal apoptosis at the injured area of the spinal cord. Besides, it has been shown that loading miRNA into the exosome structure can easily induce some processes such as promoting cancer cell apoptosis, regulating lipid metabolism, and promoting angiogenesis [114].

#### Exosomes as carriers for miRNA delivery

miRNA is a small molecule with a low molecular weight that, in addition to being easily transfected into the exosome, remains stable inside it and is, therefore, able to travel long distances without being degraded by nucleases in the blood [115]. It has been shown that by loading miRNA into the exosome structure, processes such as promoting cancer cell apoptosis, regulating lipid metabolism and promoting angiogenesis can be easily induced [116]. Ohno et al. delivered the GE11-targeted exosomes containing miRNA let-7a to EGF-overexpressing breast tumors in mice. GE11-targeted exosomes showed higher tumor suppression than control. Furthermore, the donor cells-derived exosomes transfected with the miRNA suppressed cancer cells [72]. It was reported that miR-122 overexpression in exosomes obtained from adipose MSCs can increase chemotherapy sensitivity and inhibit carcinoma growth in the mouse model [117]. Similarly, miR146b-enriched exosome is capable of suppressing EGFR and prevents tumor growth in glioma rats [118]. Moreover, the miR-451/144-enriched cardiac progenitor cell-derived exosomes promoted cardioprotection through enhancing cardiomyocyte survival in H9c2 cells, in vitro*,* and in the myocardial reperfusion/ischemia model in vivo [119].

Moreover, other forms of RNAi, such as miRNA inhibitors and miRNA mimics can be loaded into the EMVs. Mahati et al. displayed that (ScFv)-modified exosomes derived from human cord blood MSCs can be used to deliver the anti-tumor effect miR-26a mimics into the GPC3-positive hepatocellular carcinoma cells with no side effects. [120]. Exosomal miRNA delivery would be especially useful when several processes are targeted, as in the case of cancers [121, 122] and Alzheimer’s disease (miR29) [123].

#### Exosomes as carriers for mRNA delivery

Exosome-mediated mRNA delivery for COVID-19 vaccination has been recently reported. Tsai et al. validated the application of exosome for delivering the intended mRNA into the host cell in vivo and in vitro, and further specifically, developed the LSNME-SW1 vaccine, which activated broad immunities to COVID-19 [124]. Lipid nanoparticles (LNP) are currently considered one of the safest and most efficient delivery systems for mRNA, and this lipid composition has been employed in Pfizer-BioNTech and Moderna mRNA vaccines. However, some recent studies have claimed that mRNA-loaded exosomes are much more efficient than mRNA-loaded LNPs in delivering mRNA to target cells in vitro [52, 124, 125]. Moreover, it was found that mRNA-harboring exosomes can increase survival, enhance tumor inhibition, and restore tumor suppressor function in PTEN-deficient glioma mice [68]. In another study, it was shown that exosomes loaded with engineered mRNA translationally activated by corresponding miRNAs in the target cells could increase efficacy while reducing off-target uptake [74]. The results mentioned above propose that exosome is an essential tool for gene delivery with an acceptable safety profile than those of polymer-based particles, cationic lipids, and viral vectors [126].

### Challenges in RNA delivery by exosomes

The major challenges in RNA-based therapies are the instability of the RNA structure in blood circulation due to their rapid removal by nucleases and their high immunogenicity; [134], to overcome this problem, a wide variety of nanocarriers have been introduced [135]. Some delivery vehicles, such as polymers, could not be developed due to toxicity, [136], while others, such as liposomes, have not been very efficient in vivo due to poor loading efficiency and ability to penetrate the cell [137, 138]. Lipid nanoparticles (LNPs) with low toxicity and extremely high efficiency in delivering mRNA to the target cell have recently piqued the interest of all researchers [139, 140]. Exosomes, on the other hand, appear to be serious competitors for LNPs, according to some evidence [52, 125]. Because of their good biocompatibility, low immunogenicity, and ability to cross biological barriers such as the BBB, they offer many advantages as RNA delivery vehicles [141, 142]. Despite all of the benefits, the large-scale production and purification of exosomes, as well as the standard and efficient method for RNA loading, are extremely difficult and significant concerns.

#### Large scale exosome production

One of the main challenges in using exosomes in the clinic is the lack of access to an approved method for large-scale cell culture and production of exosomes with constant characteristics and properties. The presence of exosomes in the serums used in the cell culture media and the effect of its removal on the cell proliferation and consequently the property and biology of cell-derived exosomes has caused severe problems in the mass production of exosomes [143, 144].

#### Exosome purification

Almost all exosome purification methods commonly used today have either poor purity, such as methods based on volume-excluding polymers due to co-precipitation of other non-exosomal contaminants, or low yields and high costs, such as ultracentrifugation and immunoaffinity-based methods. There is no perfect purification technique today based on purity and scalability. Thus, it is another major problem in using exosomes as delivery systems [143]. Recently, there have been hopes for using the tangential flow filtration (TFF) method for reproducible large-scale exosome purification [145]. Therefore, an efficient method and good manufacturing practice (GMP) guidelines should be developed for exosomes purification [144].

#### Exosome analysis

Very precise and reliable techniques are essential for characterization and detection to assure the quality of the delivery system. The analysis and characterization process of exosomes face significant obstacles due to their small size (40 to 200 nm) and insufficient information about their structure and surface components [52, 146].

#### Long RNA loading

Exosomes are very successful in delivering miRNA and siRNA, unlike mRNA, which is very difficult to be loaded in the exosome due to their length and charge. Some mRNA loading methods were mentioned in the present study, but further studies should be performed to achieve more satisfactory and practical results in loading mRNA in exosomes [52, 147].

## Conclusion

Exosomes, a subset of extracellular vesicles, offer several benefits as drugs/biomolecules carriers over other drug nanocarriers, including the capability of loading different cargos and crossing impermeable biological barriers, facilitation of the cellular internalization of the cargos via endocytosis or membrane fusion, long half-life and circulation time, low immunogenicity, and small size [4]. These bio-inspired nanocarriers can deliver many cargos like proteins, small molecules, and nucleic acids such as DNA, siRNA, miRNA, and mRNA [22]. mRNAs play an essential role in the disease treatment and vaccine production and exosomes, owing to unique properties, can open up a new way for researchers in the development of a safe and efficient mRNA delivery system [148]. Although abundant evidence shows that lipid nanoparticles (LNPs) are highly efficient for mRNA transfer, some studies show that exosomes are more stable and less immunogenic than LNPs [52]. Recent in vitro studies have shown that transferring the CRISPR/Cas9-based RNA reporter system by exosomes has been much more satisfactory than the LNP [125]. However, due to the various challenges of using exosomes in the clinic, this extremely stable and efficient nano-carrier has not been employed in treatments yet [144]. One of the major problems is the lack of a standard and reliable method for exosome purification [149]. Although several strategies have been performed to load short nucleic acids like siRNA, shRNA, and DNA into exosomes [66, 68, 71, 72, 74], the efficient encapsulation of large mRNAs into exosomes remains a challenge. Therefore, the establishment of large-scale RNA-loaded exosomes for clinical applications should be given further attention.

## Data Availability

Not applicable.

## Abbreviations

AAT: Alpha-1-antitrypsin
AATD: Alpha-1 antitrypsin deficiency
CD: Cytosine deaminase
Dox: Doxorubicin
EPO: Encoding erythropoietin
ExoPAC: Exosomes for oral delivery of paclitaxel
EVs: Extracellular vesicles
5-FC: 5-Fluorocytosine
2FdU: 2ʹ Fluoro-deoxyuridine
5-FU: 5-Fluorouracil
HepG2: Human hepatic cell line
5hmC: 5-Hydroxymethylcytidine
IVT: In vitro-transcribed mRNA
imDCs: Immature dendritic cells
LGP2: Laboratory of genetics and physiology 2
MVBs: Multi-vesicular bodies
MDA5: Melanoma differentiation-associated protein 5
5moC: 5-Methoxycytidine
MPV: Mean platelet volume
mRNA: Messenger RNAs
MSCs: Mesenchymal stem cells
MVs: Microvesicles
PLT: Platelet counts
PRRs: Pattern recognition receptors
RBCs: Red blood cells
RBCEVs: RBC extracellular vesicles
RIG-1: Retinoic acid-inducible protein 1
RLRs: RIG-I-like receptors
TFs: Transcription factors
TLR: Toll-like receptor
UPRT: Uracil phosphoribosyltransferase
UTR: Untranslated region
VEGF-A: Vascular endothelial growth factor-A
